# Relationships between Pathological Patterns and Quality of Life: Pathway Analysis

**DOI:** 10.1155/2013/453096

**Published:** 2013-10-29

**Authors:** Shin-Woong Cho, Young-Jae Park, Sang-Chul Lee, Jaemin Ryu, Young-Bae Park, Hwan-Sup Oh

**Affiliations:** ^1^Interdisciplinary Programs, Department of Human Informatics of Oriental Medicine, Kyung Hee University, Seoul 130-701, Republic of Korea; ^2^Department of Diagnosis and Biofunctional Medicine, College of Korean Medicine, Kyung Hee University, Seoul 130-701, Republic of Korea; ^3^Department of Diagnosis and Biofunctional Medicine, Kyung Hee University Hospital at Gangdong, Seoul 134-727, Republic of Korea; ^4^Department of Business Administration, Korea Christian University, Seoul 157-722, Republic of Korea; ^5^Department of Mechanical Engineering, Kyung Hee University, Yongin, Gyeonggi-do 446-701, Republic of Korea

## Abstract

*Purpose.* The purpose of our study was to examine the relationships between pathological patterns and self-rated quality of life (QoL). *Methods.* A total of 426 Korean subjects participated in this study (male : female = 154 : 272). The subjects were asked to complete Yin Deficiency (YD), Qi Deficiency (QD), Food Stagnation (FS), Blood Stasis (BS), Phlegm, and Seven Emotions Impairment (SEI) pattern Questionnaires and the General Health Questionnaire (GHQ). We formed a pathway model consisting of pathological patterns and QoL questionnaire scores and examined which pathological patterns resulted in decreased QoL using path analysis. *Results.* Our pathway model had satisfactory fitness levels (GFI = 0.975, NFI = 0.984, and CFI = 0.984) and showed that Phlegm and SEI patterns directly resulted in decreased QoL, whereas QD, YD, FD, and BS indirectly resulted in decreased QoL. The pathway model suggested that the severity or stage of decreased QoL may be estimated by pathological patterns: QD and YD patterns are associated with the early stage; FS and BS patterns are associated with the middle stage; Phlegm and SEI are associated with the later stage of decreased QoL. *Conclusion.* Our results suggest that pathological patterns directly or indirectly affect decreases in QoL.

## 1. Introduction

In East Asian medicine (EAM), the notion of pathological patterns refers to the cause, nature, and location of pathogens at a certain stage of disease or condition of healthy Qi, Blood, Yin, or Yang affected by those pathogens [1]. In addition to being used for diagnostic purposes, pattern diagnosis guides the practitioner toward a treatment principle, whereby specific acupoints and herbal formulae are selected for treatment, using a holistic approach [2]. According to the results of pattern diagnosis, specific treatments can be prescribed to maximize effectiveness [3–5]. When conducting pattern diagnosis, self-reported symptoms are the main clues used for determining pathological patterns. For example, symptoms of fatigue and a feeling of heaviness in the limbs are important clues for determining a deficiency pattern of the Qi. However, these symptoms are subjective and difficult to evaluate objectively. Therefore, a few pathological pattern questionnaires including the Yin Deficiency Questionnaire (YDQ) [6], Qi Deficiency Questionnaire (QDQ) [7], Food Stagnation Questionnaire (FSQ) [8], Blood Stasis Questionnaire (BSQ) [9], Phlegm Pattern Questionnaire (PPQ) [10], and Seven Emotions Impairment Questionnaire (SEIQ) [11] were developed based on self-reported symptoms.

It is interesting that quality of life (QoL) is evaluated mainly by self-rated symptoms, similarly to pathological pattern questionnaires. QoL is an individual's perception of his or her position in life in the context of the culture and value systems in which he or she lives and in relation to his or her goals, expectations, standards, and concerns [12]. QoL is a broadly ranging concept, comprised of domains such as emotional well-being, social functioning, physical health, patient environment and personal beliefs, and can be assessed with both generic and condition-specific instruments [13]. QoL is particularly useful as an outcome measure in studies of diseases that have no obvious biological or clinical markers [14]. In these conditions, alleviation of symptom becomes a treatment priority, and, in the absence of objective clinical criteria, treatment efficacy should be evaluated by self-reported symptoms as well as based on patient well-being and functioning [15–17]. QoL generally correlates well with symptom severity scores in untreated patients, and improvement in QoL consequent to effective treatment has been reported [18]. The General Health Questionnaire (GHQ) is a measure of current mental health and has been widely used in different settings and different cultures [19]. As GHQ score increases, psychological QoL level decreases.

Many studies have addressed the relationships between QoL and frequently manifesting symptoms such as pain [20–22], fatigue [23], displeasure [24], disorder of emotions [25–27], and indigestion [28]. These symptoms are also associated with pathological patterns and serve as items on the pathological pattern questionnaires. Therefore, it is plausible that pathological pattern questionnaire scores are related to the QoL level. However, few studies have addressed the relationship between QoL and pathological patterns.

Cho et al. reported that SEIQ subscale scores extracted by principal component analysis (PCA) were associated with QoL, rated on a visual analog scale (VAS) [29]. However, to our knowledge, there are no studies that examined the relationships between pathological pattern scores and self-rated QoL. In this study, we used path analysis to examine these relationships. Path analysis is a straightforward extension of multiple regression models. Its aim is to provide estimates of the magnitude and significance of hypothesized causal connections between sets of variables [30]. Using path analysis, both the direct effects and indirect effects of pathological patterns on self-rated QoL can be estimated.

We formed a pathway model consisting of six pathological patterns and QoL questionnaire scores and hypothesized that the pattern scores would directly or indirectly affect the self-rated QoL scores. We examined the model-to-data fitness of our model and its effective power of the pathological patterns on QoL using path analyses.

## 2. Subjects and Methods

### 2.1. Subjects

A total of 426 Korean outpatients visiting four EAM clinics between July and August 2011 for pain-related problems including shoulder pain, low back pain, and ankle sprain, as well as headache, dizziness, indigestion, and chronic fatigue problems, participated in this study (male : female = 154 : 272).  Table 1 lists the age distribution of subjects by gender. After the purpose of this study was presented to the subjects and informed consent was obtained from all subjects, they were asked to complete YDQ, QDQ, FSQ, BSQ, PPQ, SEIQ, and GHQ questionnaires.  Table 2 lists questionnaire items of the YDQ, QDQ, FSQ, BSQ, PPQ, and SEIQ.

### 2.2. Questionnaires

To estimate decreases in QoL, we used the Korean version of the GHQ (K-GHQ) [31]. The K-GHQ consists of 28 items and is categorized into four domains: somatic symptoms, anxiety/insomnia, self-dysfunction, and severe depression. As mentioned in the introduction, increased total score of the GHQ leads to decreased QoL. Each item of the K-GHQ was rated on a 4-point Likert scale: 0 = not at all; 1 = no more than usual; 2 = rather more than usual; 3 = much more than usual. The GHQ scoring system transforms Likert scores of 0 and 1 to 0 points and Likert scores of 2 and 3 to 1 point. This dichotomous scoring method has the advantage of eliminating errors due to end users and middle users. Therefore, the 28 dichotomous total scores of the K-GHQ were summed in this study.

Yin Deficiency (YD) is a pattern resulting from deficiency of Yin fluid and essence and usually manifests as emaciation, dizziness, tinnitus, dryness of the mouth and throat, afternoon fever, and night sweats [1]. YDQ consists of 30 items related to YD [6]. The QDQ is a 22-item questionnaire relating to spleen Qi Deficiency (QD) [7]. Food Stagnation (FS) manifests as epigastric and abdominal distention, dyspepsia, water brash, anorexia, offensive odor of stools, and curd-like and slimy tongue coating [1]. The FSQ consists of 20 items to estimate symptoms related to FS [8]. Blood Stasis (BS) is defined as a pathological product of blood stagnation, including extravasated blood and sluggishly circulating blood or blood congested in a viscus, all of which may result in pathology [1]. The BSQ consists of 14 items to estimate symptoms related to BS [9]. Phlegm is the viscous turbid pathological product that can accumulate in the body, causing a variety of diseases [10]. The PPQ consists of 26 items to estimate self-rated symptoms related to Phlegm Pattern (PP) [10]. Seven Emotions Impairment (SEI) is a collective term for joy, anger, thought, anxiety, sorrow, fear, and fright, taken as endogenous factors causing diseases if in excess [1]. The SEIQ consists of 22 items to evaluate SEI [11]. The YDQ, QDQ, FSQ, BSQ, PPQ, and SEIQ were rated on a 7-point Likert scale: 1 = disagree very strongly; 2 = disagree strongly; 3 = disagree; 4 = neither agree nor disagree; 5 = agree; 6 = agree strongly; and 7 = agree very strongly. When conducting path analysis, the total scores of the six pattern questionnaires were subtracted from the number of total items to moderate the mean values.

### 2.3. Research Model

**Figure 1:**
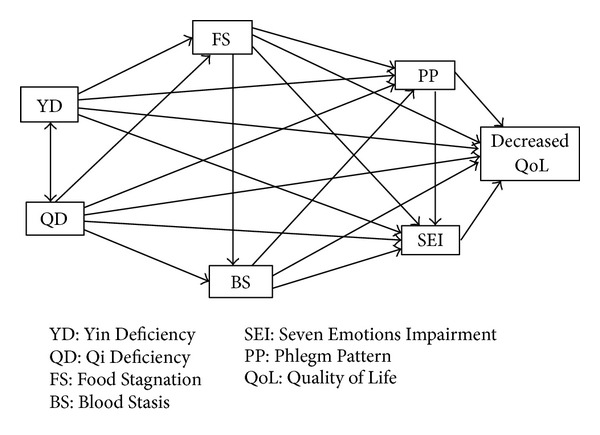
Research model for relationships between pathological patterns and quality of life.

The relationships between the six pathological patterns were formed based on the EAM theory; the research model is depicted in Figure 1. YD affects FS because fluid, a source of Yin, is necessary when digesting food [32]. Deficiency fire resultant from YD burns and concentrates fluid and may form Phlegm [33]. Deficiency fire resultant from YD occurs in the upper body and results in palpitation, insomnia, and burning in the chest. Therefore, YD may affect SEI [32]. 

Digestion is conducted by spleen Qi, and QD may affect FS, which manifests as dyspepsia [32]. It is generally accepted that fluid cannot be transported to all parts of the body in the QD condition, and it may be deformed to Phlegm [32]. Spleen QD and heart QD are the main factors of SEI [33]. Blood circulates through the meridians with the assistance of the Qi. However, blood may stagnate in QD conditions, and BS may occur [32].

FS and BS have some related symptoms including abdominal lumps, nosebleeds, and bloody stools. FS also shares some SEI-related symptoms such as flank pain, chills and fever, and chest pain. These symptoms may be due to the effects of FS on BS and SEI [33–35]. FS, like QD, results in deformation of the fluid and may affect the formation of Phlegm. BS facilitates the formation of tumors or lumps, which in EAM theory are considered to be combinations of BS and Phlegm [33]. Therefore, it is possible that BS may affect PP. BS affects emotions, especially in women. For example, BS in the lower abdomen during menstruation may result in severe irritability [33, 36]. Thus, BS may affect SEI. Finally, Phlegm stagnated in the upper body results in neurophysiological symptoms such as palpitation, dizziness, chest discomfort, and sensitivity to noise [9]. Therefore, PP may affect SEI [33]. After forming the 12 pathways consisting of the six pathological patterns, the six direct pathways of the pathological patterns for decreased QoL estimated by GHQ scores were added to the pathway model.

### 2.4. Statistical Analysis

In our study, path analysis was used to examine whether the pathway model was acceptable and to examine which pathways among direct and indirect effects were more influential on QoL. We calculated three model fitness indexes, Goodness-of-Fit Index (GFI), Normed Fit Index (NFI), and Comparative Fit Index (CFI), to strictly evaluate the fitness of the proposed model [37–40]. Standardized estimates were calculated to compare the relative predictive powers of pathological patterns to decreases in QoL. Squared multiple correlations (SMC) were calculated to examine the percentage of pathological patterns contributing to decreases in QoL or were explained by other pathological patterns. All statistical analyses were performed with AMOS 18. In the model fitness tests, GFI > 0.950, 0 < NFI < 1, and CFI > 0.90 indicated statistical significance [37, 41, 42]. In examining standardized estimates of the direct and indirect effects, *P* < 0.05 indicated statistical significance. 

## 3. Results

**Figure 2:**
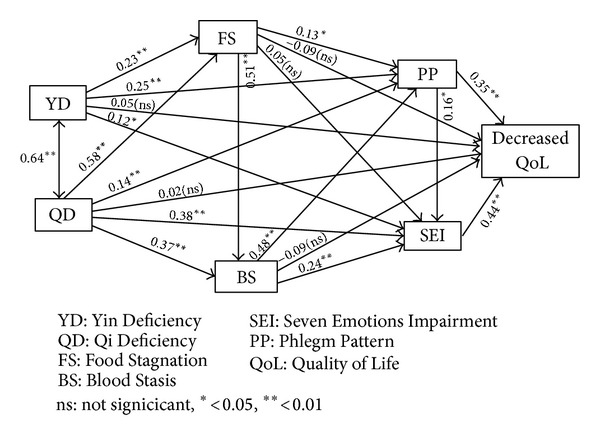
Results of path analysis. The model provided a good fit (GFI = 0.975, NFI = 0.984, CFI = 0.984). Note that the correlation between YD and QD is located on a two-directional arrow, whereas standard estimates of each pathological pattern to other patterns or QoL are located on the corresponding one-directional arrows.

The results of the research model are presented in Figure 2. Correlation appears as a two-directional arrow, and standardized path coefficients appear as one-directional arrows. Three fitness indices showed that our research model possessed a satisfactory model-to-data fitness level (GFI = 0.975, NFI = 0.984, and CFI = 0.984).

Table 3 lists SMC and standardized estimates of the pathological patterns and decreases in QoL. The SMC values of FS, BS, Phlegm, SEI, and K-GHQ were 0.555, 0.667, 0.775, 0.726, and 0.437, respectively, indicating that Phlegm and SEI patterns result from other pathological patterns. Decreases in QoL were significantly affected by two pathological patterns: SEI (*β* = 0.444) and Phlegm patterns (*β* = 0.350). The highest estimate for the standardized regression weight was found in the path from the QD to FS (*β* = 0.576), and both the path from FS to BS (*β* = 0.507) and the path from BS to Phlegm (*β* = 0.481) showed higher estimates than other pathways.

Table 4 lists direct, indirect, and total standardized estimates of the pathological patterns and decreases in QoL. YD, QD, BS, PP, and SEI all showed significant total effects (*β* = 0.233, 0.389, 0.220, 0.423, and 0.444, resp.). Interestingly, YD, QD, and BS pathological patterns had only indirect effects on decreases in QoL. QD was a strong determinant of the FS, BS, Phlegm, and SEI patterns (*β* = 0.576, 0.664, 0.537, and 0.658, resp.). FS was a strong effector of BS (*β* = 0.507), indicating that QD has an indirect effect on decreases in QoL via other pathological patterns such as BS, FS, SEI, or PP.

## 4. Discussion

In this study, we hypothesized that pathological patterns would affect decreases in QoL, and we formed a pathway model according to the EAM theory. Our pathway model had satisfactory model-to-data fitness levels (GFI = 0.975, NFI = 0.984, and CFI = 0.984). As hypothesized, pathological patterns affected decreases in QoL. The influence of pathological patterns on decreases in QoL could be classified into direct and indirect pathological pattern determinants. For example, Phlegm and SEI directly affected decreases in QoL, and the total path estimates of the two patterns were similar, indicating that progressive Phlegm and SEI patterns directly induce decreases in QoL. Interestingly, the total effects of SEI on QoL decrease consist of only direct effects, whereas those of Phlegm consist of both direct and indirect effects, suggesting that the Phlegm pattern has a direct effect on decrease in QoL or an indirect effect on the aggravation of SEI followed by a decrease in QoL.

It is noteworthy that QD, YD, and BS patterns only indirectly affected decreases in QoL. For example, QD and BS patterns resulted in the formation or progression of PP and SEI, and, thereafter, formed or progressive Phlegm and SEI patterns decreased QoL. Especially, QD was a strong and general effector of the BS, Phlegm, SEI, and FS patterns. Although YD was also a general effector of the Phlegm, SEI, BS, and FS patterns, its efficacy was not as strong as that of QD. It is interesting that FS induces an increase in the Phlegm, BS, and SEI patterns, although it had a weak indirect effect on decreases in QoL, suggesting that FS, like other pathological patterns, should be considered and managed for the purpose of improving QoL. According to our pathway model, QD and YD patterns should be preferably estimated and managed in order not to result in decreases in QoL. When QD and YD were not managed, the FS or BS pattern occurred. In this stage, FS and BS formed secondarily from QD and YD induced Phlegm or SEI formation, which directly lowers the QoL. Taken together, our study results suggest that pathological patterns can be categorized into three levels from the QoL-etiological perspective: increased QD and YD patterns refer to the early stage of decreases in QoL, FS and BS patterns refer to the middle stage, and increases in Phlegm and SEI patterns refer to the latter stage of decreases in QoL. Therefore, in individuals with Phlegm or SEI patterns, QoL issues should be more intensively managed.

In summary, we hypothesized that pathological patterns may affect decreases in QoL and form a pathway model consisting of YDQ, QDQ, FSQ, BSQ, PPQ, SEIQ, and K-GHQ scores. Our pathway model had satisfactory model-to-data fitness level and suggested that pathological patterns could be categorized into direct and indirect pathological pattern determinants of QoL decrease. Moreover, the severity or stage of QoL problems could be estimated by the pathological patterns, and therefore, suitable treatments to alleviate each pathological pattern can be conducted to improve QoL. Although, in this study, we proposed a pathway model with a satisfactory model-to-data fitness and presented the causalities of pathological patterns to decreases in QoL, our study had some limitations. First, our study was conducted only in Seoul, Korea, and, as a result, has limited population validity. Second, GHQ has the inherent limitation of examining QoL only from the psychological point of view. Further studies are required in order to overcome these limitations with respect to population validity and psychological QoL.

## 5. Conclusions

In this study, we hypothesized that pathological patterns would affect QoL. We formed a pathway model consisting of YDQ, QDQ, FSQ, BSQ, PPQ, SEIQ, and K-GHQ scores according to the EAM theory and examined the model-to-data fitness of our pathway model; our proposed pathway model had satisfactory fitness levels (GFI = 0.975, NFI = 0.984, and CFI = 0.984). The pathway model showed that Phlegm and SEI patterns were direct effectors on QoL, whereas QD, YD, FD, and BS were indirect effectors on QoL. The pathway model results also suggested that the severity or stage of decreased QoL could be estimated by the pathological patterns: QD and YD patterns were linked to the early stage; FS and BS patterns were linked to the middle stage; Phlegm and SEI patterns were linked to the latter stage of decreased QoL. A suitable pattern treatment should be conducted to improve QoL according to stage. Further studies are required in order to overcome the study limitations with respect to population validity and psychological QoL.

## Figures and Tables

**Table 1:** Age distribution of the participants.

Age	Male (*n*)	Female (*n*)	Total (*n*)
20–29	37	42	79
30–39	33	48	81
40–49	24	51	75
50–59	39	85	124
60–64	21	46	67

Total	154	272	426

**Table 2:** Yin Deficiency, Qi Deficiency, Food Stagnation, Blood Stasis, Phlegm, and Seven Emotions Impairment Questionnaires items.

Yin Deficiency Questionnaire (*n* = 30)	Qi Deficiency Questionnaire (*n* = 22)	Food Stagnation Questionnaire (*n* = 20)	Blood Stasis Questionnaire (*n* = 14)	Phlegm Pattern Questionnaire (*n* = 26)	Seven Emotions Impairment Questionnaire (*n* = 22)
I urinate frequently	I usually feel tired or languid	I feel pain in the pit of my stomach	I recently sprained my ankle or waist	I feel unclear in the head	I am often angry
My urine is dark yellow	I feel heavy or weak in the limbs	I have a feeling of fullness in the stomach after eating	I recently was hurt in a fall or traffic accident	I have a headache	I have alternating chills and fever
I feel residual urine	I have trouble standing or walking for a long time	I often have an upset stomach	I was operated on ( ) times	I feel dizzy	I feel dizzy
I cannot contain my urine	I have a heavy feeling in my anus	I feel stomach pain immediately after eating	I have dull pain that lasts for a long time	I have ringing in the ears	I feel heavy in the chest
I wake to urinate in the night	My memory has gone from bad to worse	I often belch	I have joint pain	I feel my heart palpitates	I have tightening in the chest
My stool is hard	I often catch common colds	I have water brash	I have lower abdominal pain	I am startled by faint noise	I have chest pain
My hair falls out	My colds last for a long time	I feel sick to my stomach	I have flank pain	I feel heavy in the chest	I often sigh
I have a rough skin	My voice easily becomes hoarse after talking	I have a bowel movement immediately after eating	I have pain that disturbs my sleep in the night	I have a cough	I taste bitter tastes
I have an itch in the night	I often have a weak voice	I am very fond of eating	I often have a bruise	I have sputum in my throat	I feel my heart palpitates
I have a dry mouth	I sweat spontaneously	I feel heavy in the body	My lips or tongue is dark blue	I feel a foreign body present in the throat, neither swallowed nor ejected	I am sleepless
My heel is dry and cracked	I often have a fever	I feel languid after eating	My face is dark blue	I feel short of breath	I am forgetful
I do not gain weight despite eating fully	I often experience nosebleeds	I swell in the face or limbs	I have dark circles under the eyes	I feel fatigued	I am startled by faint noise
I have a fever in the night	I overwork myself	I gained weight recently	My stool is black	I feel heavy or weak in the limbs	I feel down and uninterested in everything
I have a fever in the afternoon	My work hours are irregular	I often urinate	I feel a lump in my abdomen	I have a poor appetite	I feel like lying because of fatigue
I have a flush in the afternoon	I am under stress because of my work	My stool is mucousy		I feel sick to the stomach	I have indigestion despite a normal appetite
My soles are hot in the night	I feel pain after working	I have pain in the joints		I have indigestion	I feel uneasy
I feel hot deep in the body, for example, in the bone	I feel short of breath after working	I have a water-change-related diarrhea or abdominal pain		I have a feeling of fullness in the stomach with just a little food	I sweat during sleep
I prefer cold beverages to warm beverages	My mealtimes are irregular	I have a food-related allergy		My stomach or intestine rumbles	I have many things worrying me
I am susceptible to heat and cold	I feel weak after skipping meals	I have abdominal fullness or diarrhea after drinking		My stool is mucousy	I have trouble with my family
I sweat during sleep	I feel drowsy or languid after meals	(for women) I have vaginal discharge		I have a lump somewhere on my body	I have vaginal bleeding during sex
My ear rings	I have indigestion			My face is yellowish	My menstrual bleeding volume is irregular
I have a cough in the afternoon	I have a poor appetite			I have dark circles under the eyes	My menstrual period is irregular
I have a cough in the night				I feel itchy	
My cough lasts for a long time				I have pain in the joints	
I feel tired or languid				I have flank pain	
I feel tired in the morning				I gained or lost weight recently	
I feel low back pain					
I feel dull pain in my ankle or knee					
I feel heavy or weak in my lower limbs					
I feel dull pain in my heel					

**Table 3:** Results of path analysis.

Regression weight or correlation	Pathological pattern or QoL	SMC	Standardized estimate	Estimate	S.E.	C.R.	*P* value
Regression weight	Yin Deficiency → Food Stagnation	0.555	0.230	0.266	0.049	5.457	<0.001
	Qi Deficiency → Food Stagnation		0.576	0.436	0.032	13.668	<0.001
	Food Stagnation → Blood Stasis	0.667	0.507	0.449	0.036	12.500	<0.001
	Qi Deficiency → Blood Stasis		0.372	0.250	0.027	9.173	<0.001
	Food Stagnation → Phlegm	0.775	0.129	0.135	0.042	3.236	0.001
	Blood Stasis → Phlegm		0.481	0.564	0.047	12.037	<0.001
	Yin Deficiency → Phlegm		0.253	0.305	0.037	8.145	<0.001
	Qi Deficiency → Phlegm		0.143	0.113	0.031	3.682	<0.001
	Yin Deficiency → Seven Emotions Impairment	0.726	0.121	0.174	0.053	3.284	0.001
	Food Stagnation → Seven Emotions Impairment		0.052	0.065	0.055	1.168	0.243
	Qi Deficiency → Seven Emotions Impairment		0.382	0.359	0.041	8.762	<0.001
	Blood Stasis → Seven Emotions Impairment		0.239	0.335	0.071	4.692	<0.001
	Phlegm → Seven Emotions Impairment		0.163	0.195	0.064	3.052	0.002
	Seven Emotions Impairment → quality of life	0.437	0.444	1.739	0.273	6.379	<0.001
	Qi Deficiency → quality of life		0.019	0.072	0.250	0.287	0.774
	Blood Stasis → quality of life		−0.089	−0.489	0.411	−1.190	0.234
	Food Stagnation → quality of life		−0.088	−0.430	0.312	−1.379	0.168
	Yin Deficiency → quality of life		0.050	0.281	0.301	0.935	0.350
	Phlegm → quality of life		0.350	1.637	0.362	4.521	<0.001

Correlation	Yin Deficiency *↔* Qi Deficiency		0.641	0.511	0.046	11.122	<0.001

SMC: squared multiple correlations, S.E: standard error, C.R.: critical ratio.

**Table 4:** Results of total, direct, and indirect effects.

Dependent variable	Standardized effect	Independent variable					
		Qi Deficiency	Yin Deficiency	Food Stagnation	Blood Stasis	Phlegm	Seven Emotions Impairment
Food Stagnation	Total	0.576*	0.230*				
	Direct	0.576*	0.230*				
	Indirect	0.000	0.000				

Blood Stasis	Total	0.664*	0.117**	0.507**			
	Direct	0.372*	0.000	0.507**			
	Indirect	0.292*	0.117**	0.000			

Phlegm	Total	0.537**	0.339*	0.373**	0.481**		
	Direct	0.143**	0.253*	0.129*	0.481**		
	Indirect	0.394*	0.086**	0.244**	0.000		

Seven Emotions Impairment	Total	0.658**	0.216*	0.234*	0.318**	0.163*	
	Direct	0.382**	0.121*	0.052	0.239*	0.163*	
	Indirect	0.276*	0.095*	0.182**	0.078*	0.000	

Decreases in quality of life	Total	0.389**	0.233*	0.101	0.220**	0.423*	0.444*
	Direct	0.019	0.050	−0.088	−0.089	0.350*	0.444*
	Indirect	0.370**	0.184**	0.189**	0.309**	0.072**	0.000

*P < 0.05, **P < 0.01.

## Abbreviations

EAM: East Asian medicine
YDQ: Yin Deficiency Questionnaire
QDQ: Qi Deficiency Questionnaire
SEIQ: Seven Emotions Impairment Questionnaire
PPQ: Phlegm Pattern Questionnaire
FSQ: Food Stagnation Questionnaire
BSQ: Blood Stasis Questionnaire
QoL: Quality of life
GHQ: General Health Questionnaire
PCA: Principal component analysis
VAS: Visual analog scale
K-GHQ: The Korean version of the GHQ
YD: Yin Deficiency
QD: Qi Deficiency
SEI: Seven Emotions Impairment
FS: Food Stagnation
BS: Blood Stasis
PP: Phlegm Pattern
GFI: Goodness-of-fit Index
NFI: Normed fit index
CFI: Comparative fit index
SMC: Squared multiple correlations.
